# Safety and Efficacy of an Oncolytic Adenovirus as an Immunotherapy for Canine Cancer Patients

**DOI:** 10.3390/vetsci9070327

**Published:** 2022-06-28

**Authors:** Clara Martín-Carrasco, Pablo Delgado-Bonet, Beatriz Davinia Tomeo-Martín, Josep Pastor, Claudia de la Riva, Paula Palau-Concejo, Noemí del Castillo, Javier García-Castro, Ana Judith Perisé-Barrios

**Affiliations:** 1 Small Animal Surgery Service, Hospital Clínico Veterinario, Universidad Alfonso X el Sabio, 28691 Madrid, Spain; ccarrmar@uax.es; 2 Unidad de Investigación Biomédica (UIB-UAX), Universidad Alfonso X el Sabio, 28691 Madrid, Spain; pdelgbon@uax.es (P.D.-B.); btomemar@uax.es (B.D.T.-M.); paulapalau@hotmail.es (P.P.-C.); 3 Animal Medicine and Surgery Department, Fundació Hospital Clínic Veterinari, Universitat Autònoma de Barcelona, 08193 Cerdanyola del Vallès, Spain; josep.pastor@uab.cat; 4 Oncology Service, Hospital Clínico Veterinario, Universidad Alfonso X el Sabio, 28691 Madrid, Spain; cdelafra@uax.es (C.d.l.R.); noemidelcastillom@gmail.com (N.d.C.); 5 Cellular Biotechnology Unit, Instituto de Salud Carlos III, 28220 Madrid, Spain; jgcastro@isciii.es

**Keywords:** oncolytic virus, virotherapy, immunotherapy, ICOCAV15, canine carcinoma

## Abstract

**Simple Summary:**

The use of oncolytic virus is an innovative approach that has shown promising results as a treatment in oncology. Epithelial-derived tumors are the most frequent neoplasms in dogs, but gold standard therapies can be highly invasive procedures. Due to the accessible localization of these tumors, the intratumoral administration is feasible. Therefore, we propose to determine the safety and efficacy of intratumoral administration of oncolytic adenovirus ICOCAV15, in canine patients with epithelial-derived tumors. Eight dogs with carcinoma/adenocarcinoma were intratumorally treated with ICOCAV15. No clinically relevant changes were observed in the blood count, biochemistry and coagulation test analyzed during follow-up. The survival time of the 6/8 dogs exceeded the median survival time with chemotherapy, showing a partial response rate of 25% and 75% of stable disease. ICOCAV15 was detected in the target lesion by qPCR and immunohistochemistry. Also, some of the non-treated metastasis showed an infiltration of ICOCAV15 by immunohistochemistry. The immune populations were evaluated, and an increase of CD8+, MAC387+, CD3+ and CD20+ cells was reported in some of the patients after the inoculation. These results show that intratumoral ICOCAV15 is safe and well tolerated by dogs. Also, they suggest ICOCAV15 could be a new tool in veterinary oncology for accessible carcinomas/adenocarcinomas.

**Abstract:**

The use of oncolytic viruses is an innovative approach to lyse tumor cells and induce antitumor immune responses. Eight dogs diagnosed with carcinoma/adenocarcinoma were intratumorally treated with ICOCAV15, an oncolytic canine adenovirus (CAV). To evaluate the treatment’s safety, a blood count, biochemistry, and coagulation test were performed before treatment and during follow-up. Immune populations were analyzed by flow cytometry. Anti-adenovirus antibodies were also determined. The immune infiltration, vascularization, and viral presence in the tumor were determined by CD3, CD4, CD20, CD31 and CAV by immunohistochemistry. All the dogs maintained a good quality of life during follow-up, and some had increased median survival time when compared with dogs treated with chemotherapy. No treatment-related adverse effects were detected. The Response Evaluation Criteria In Solid Tumors criteria were also assessed: two patients showed a partial response and the rest showed stable disease at various times during the study. ICOCAV15 was detected inside the tumor during follow-up, and antiviral antibodies were detected in all patients. Furthermore, the tumor-infiltrating immune cells increased after viral administration. Therefore, we suggest that intratumorally administered ICOCAV15 could represent as a new tool for the treatment of canine carcinoma because it is safe, well-tolerated by dogs, and shows promising results.

## 1. Introduction

During the last few years, the incidence of tumors in canine patients has been increasing, principally due to the increase in pets’ lifespan, and tumors have become the primary cause of death and one of the greatest challenges in veterinary medicine [1]. Cutaneous and subcutaneous origins are the most frequent neoplasms, representing one-third of all diagnosed tumors in dogs [2]. Conventional therapies to treat canine tumors have certain disadvantages due to their low specificity, surgical limitations for non-accessible tumors [3], and the low availability of radiotherapy in some countries [4,5,6,7]. Due to these issues, immunotherapy is becoming one of the main pillars of anti-tumoral treatment research [8].

The influence of the immune system on the evolution of tumor growth has been evidenced in multiple reports [9,10,11,12]. Several cells present immunosurveillance functions, such as natural killer cells and macrophages, which detect and control cancerous cells in development, or dendritic cells, which activate CD8+ T cells in the tumor microenvironment (TME) [13,14]. This mechanism controls tumor progression when mutations in tumoral cells facilitate tumor growth and further promote their dissemination, evading the immune mechanisms activated in the patient [12,13,15]. These processes that facilitate tumor spread have been demonstrated in canine and human tumors, suggesting a new therapeutic route to treat cancer [11,13]. The ability of the immune system to recognize tumors has made it a field of interest for developing new therapeutic approaches in oncology. Immunotherapy typically addresses two main pathways: stimulating an immune response against the tumor and counteracting the inhibitory mechanisms of the TME that favor tumor growth [10,16,17]. Immunotherapy is a reality in human medicine, with more than 3270 ongoing clinical trials [18], and new therapeutic options have also shown promising results in veterinary medicine [19,20]. These therapies have fewer secondary effects and present a clear advantage in veterinary oncology, given that they are more tolerable by the patient than conventional therapies, allowing the animals to maintain their quality of life [17]. Although there are many varieties of immunotherapy, the use of oncolytic viruses (OVs) is an innovative approach that has become an interesting field of research, with several promising results as an oncological treatment [21,22,23,24].

OVs are genetically modified to have a selective replication in tumor cells, and several studies have identified various mechanisms that could induce a clinical response, including cellular lysis, antitumoral immunity, and vascular collapse [21,25,26,27,28]. The approval of Imlygic a few years ago by the US Food and Drug Administration and the European Medicines Agency for human patients with melanoma facilitated and promoted new investigations with various viral therapies. Several viruses are being used in current veterinary medicine for oncolytic therapy, such as adenovirus [20], measles virus [29], distemper virus [30], herpes simplex virus [31], Newcastle disease virus [32], reovirus [33], and vaccinia virus [34,35], among others. Although many viruses have been developed, oncolytic adenoviruses (Ads) are still the most commonly used for oncolytic virotherapy due to their infection efficacy, high titer production, safety, easy genetic modification, and well-studied replication characteristics [36].

The efficacy of Ads has been proven in several murine models, and further in veterinary and human clinical trials, with the very few adverse effects limited to mild flu-like symptoms, which can be more severe after systemic administration, and local reactions at the injection site [21,22,25,27,37]. In veterinary studies of local administration, one isolated case of coagulation disturbance has been reported [38]. Although these studies have shown promising results, all of them concur that one issue that needs to be improved in virotherapy is its reduced efficacy when administered systemically. To avoid the inactivation of the viral particles, several mechanisms have been proposed, such as the use of mesenchymal stem cells, liposomes, or intratumoral inoculation [10,20,35,39].

The most frequent tumors in dogs are cutaneous and subcutaneous, of epithelial origin, and the gold-standard therapies are either not available (radiotherapy) or are highly invasive procedures (surgery) [2,3,6]. The overall incidence of tumors determined to be of epithelial origin in dogs has been reported to be from 26 to 43%, in some cases being very aggressive and invasive tumors [1,2]. These types of tumors are relatively common in daily veterinary practice, and the intratumoral administration of OV can be easy to handle in this setting. The intratumoral use of OV in human literature for similar tumors has shown an 83% response rate with minimal adverse effects, implying it could be a feasible treatment for dogs with epithelial tumors [40]. The OV ICOCAV15 is based on the canine wild-type adenovirus canine type 2 (CAV2), with an RGD motif inserted in the CAV2 knob. The modification of the endogenous E1a promoter, by the insertion of E2F-binding sites and the deletion of the pRB-binding domain (E1aD21), allows ICOCAV15 to be a conditionally replicative virus that can finish the viral cycle in cells with a disrupted retinoblastoma pathway [37,38,41]. Our hypothesis was that intratumoral ICOCAV15 can stimulate the immune environment inside the tumor to expand, produce tumor cell lysis, and improve the median survival time without causing adverse effects in dogs. Therefore, we proposed to determine the safety and efficacy of intratumoral inoculation with ICOCAV15, a canine Ad (CAV) that replicates specifically in tumor cells, given that this treatment could improve outcomes for these oncologic patients.

## 2. Materials and Methods

### 2.1. Study Design

Eight canine patients diagnosed with carcinoma/adenocarcinoma were enrolled for intratumoral treatment with ICOCAV15 at the Universidad Alfonso X el Sabio–Hospital Clínico Veterinario, Madrid, Spain (Table 1). The study was approved by the Ethics and Professional Integrity Committee of the Official College of Veterinarians of Madrid (NP 8384 in 2020). The inclusion criteria were the owners’ rejection of the surgical procedure, inoperable tumors, no response to the standard chemotherapy protocols, absence of undercurrent disease, accessible lesions, and a docile character to allow follow-up without sedation. The virotherapy was authorized as compassionate treatment; therefore, a control group with healthy animals was not included in the study. The diagnosis procedure included a whole-body computed tomography (CT) with iodized contrast (IOHEXOL, Omnipaque at 2 mL/kg) to assess staging, with a tissue biopsy for histopathology analysis. To perform the treatment procedure, the canine patient was mildly sedated with acepromazine (0.03 mg/kg) and methadone (0.1 mg/kg) to perform CT, a tissue biopsy, and the intratumoral inoculation of ICOCAV15. If the anesthesiologist required an induction of the patient for safety, thiopental (dose effect) and isoflurane (inhalant agent) were used. The ICOCAV15 was kindly provided by Ramón Alemany Bonastre (IDIBELL-Institut Català d’Oncologia, l’Hospitalet de Llobregat, Spain). The veterinary clinician determined 3–5 quadrants of the visible tumor for intratumoral inoculation with ICOCAV15 (10^7^ i.u.) diluted in saline solution (500 μL total volume), using a 23G needle. For the cases in which the inoculation had to be guided by ultrasound, the administration of the virus was distributed throughout the line of the needle path. After the inoculation of the oncolytic CAV, the patients were closely monitored for six hours to detect any early adverse effects due to the therapy. Blood analysis, tumor measurements, and clinical anamnesis were evaluated on the seventh day, one month later, and then every two months (for the first year) to detect late adverse effects, to ensure the well-being of the patient and to evaluate the tumor response according to the Response Evaluation Criteria In Solid Tumors (RECIST) criteria [42]. Tissue samples were obtained during each medical check-up for later analysis. A second dose was administered, with the owner’s consent, at least 76 days after the first dose; we followed the same procedure as for the first inoculation. Lastly, 7/8 patients received the second dose (PSit07 had died) and 2/8 received a third dose (PSit04 after 185 days and PSit06 244 days after the first dose) to evaluate possible beneficial cumulative effects.

### 2.2. Safety and Efficacy

To evaluate the possible adverse effects and any possible deterioration of health in the canine patients, we processed blood samples collected with heparin (to obtain plasma) and ethylenediaminetetraacetic acid (EDTA) (for flow cytometry analysis) tubes. We evaluated hematocrit; hemoglobin; mean corpuscular volume; mean corpuscular hemoglobin concentration; mean corpuscular hemoglobin; red cell blood distribution width; reticulocytes; neutrophils; lymphocytes; monocytes; eosinophils; basophils; platelets; mean platelet volume; platelet distribution width; ions (Mg^2+^, Na^+^, K^+^, Cl^−^, Ca^2+^, PO4^3-^); renal parameters (urea, blood urea nitrogen, creatinine); hepatic transaminases (alkaline phosphatase, aspartate aminotransferase [AST], alanine transaminase [ALT]); total proteins (albumin, globulin, and albumin/globulin ratio); coagulation times (time of prothrombin, partial time of activated thromboplastin, fibrinogen); basal glucose. In addition, at each check-up with the veterinarian, the owner completed a questionnaire (adapted from [43]) to evaluate the dog’s quality of life. The questionnaire has 12 questions with responses scored from 0 to 3 (Table S1). The maximum score is 36, and scores lower than 20 indicate a poor quality of life. The tumor response was evaluated following RECIST criteria, with the baseline measurements taken before each inoculation. Complete response: disappearance of all target lesions, pathologic lymph nodes < 10 mm on the short axis; partial response (PR): at least 30% reduction in the sum of the target lesion diameters, taking as a reference the baseline sum; progressive disease (PD): either the appearance of new lesions or a >20% increase in the sum of the target lesion diameters, taking as a reference the smallest sum in the timeline between doses (the sum must also show an absolute increase of 5 mm); stable disease (SD): less than a 30% reduction or a 20% increase in the sum of the target lesion diameters, taking as a reference the smallest sum of the diameters during the study.

### 2.3. ICOCAV15 DNA Extraction and Quantification

Biopsies obtained at each check-up, as well as the tissues obtained at necropsies, were stored as dried pieces at −80 °C until processing. We disaggregated 10 to 30 mg of each tissue, and extracted DNA with a specific commercial kit to isolate DNA from tissues (E.Z.N.A.^®^ Tissue DNA kit; Omega biotech, Norcross, GA, USA), following the manufacturer’s instructions. We analyzed the quantification and the purity of the DNA (A260/280 and A260/230) with a Nanodrop 2000 spectrophotometer (Thermo Scientific, Waltham, MA, USA). A standard curve was performed with serial dilutions of ICOCAV15 from 0.4 × 10^6^ viral particles (vp)/well to 4 vp/well. Triplicates of the purified DNA samples were analyzed by the quantitative real-time polymerase chain reaction (PCR) using the QuantStudio 3 Real-Time PCR (Applied Biosystems, Waltham, MA, USA). We used the Premix Ex Taq (Clontech Laboratories Inc, Mountain View, CA, USA), primer forward (0.5 μmol/L) 5′-TGTGGGCCTGTGTGATTCCT-3′, primer reverse (0.5 μmol/L) 5′-CCAGAATCAGCCTCAGTGCTC-3′, and 10 pmol of Taqman probe FAM-CTCGAATCAGTGTCAGGCTCCGCA-TAMRA for 40 cycles of 15 s at 95 °C and 1 min at 60 °C. We analyzed the data obtained with QuantStudio 3 software (Applied Biosystem). The Ct number detected in each well was interpolated on the standard curve to quantify the copy number of ICOCAV15 in the samples, and we calculated the mean of the triplicates.

### 2.4. Flow Cytometry

We kept cryopreserved samples for flow cytometry at −80 °C until analysis. We thawed frozen samples in a water bath at 37 °C, then centrifuged and resuspended them in phosphate-buffered saline (PBS) with 2% fetal bovine serum (FBS). We added 50 µL of cell suspension to 250 µL of PBS with 2% FBS and incubated it at room temperature for 30 min with the selected antibody panel, then washed it twice, resuspended it in PBS, and analyzed it within one hour. Data analysis collection was performed with a Cytoflex S (Beckman Coulter, Brea, CA, USA) and Cytoexpert software version 2.4.028 (Beckman Coulter). A total of 20,000 events were analyzed for each tube. Tube 1 contained 5 µg of propidium iodide for dead cell exclusion, and the live cell population was gated by the side scatter cytogram versus the forward scatter cytogram. Tube 2 had the following antibody panel: CD21-PE (Bio-Rad, Hercules, CA, USA, clone CA2.1D6) to detect B cells; CD5-FITC (Bio-Rad, clone YKIX322.3) to detect T lymphocytes (helper and cytotoxic); CD56-APC (Sysmex, Barcelona, Spain, clone LT56) to detect a cell subset with the CD56+ phenotype. Tube 3 had CD8-PE (Bio Rad, clone YCATE55.9) to detect cytotoxic lymphocytes and CD4-FITC (Bio-Rad, clone YKIX302.9) to detect T helper lymphocytes. Tube 4 had CD14-PE (Bio-rad, clone Tük4) to detect monocytes and macrophages, and class II MHC-FITC (Bio-Rad, clone YKIX334.2) to detect antigen-presenting cells (including B cells) and also activated T lymphocytes.

### 2.5. Immunohistochemistry

The canine biopsies were fixed in 10% formalin for preservation, and they were embedded in paraffin. Sections of 5 mm were cut with a microtome and dewaxed, then rehydrated using an alcohol battery (xylol 2 × 5 min, ethanol 100% 2 × 5 min, ethanol 96% 1 × 5 min, ethanol 70% 1 × 5 min) for hematoxylin–eosin staining or immunostaining. To perform the immunostaining, a previous antigen retrieval with citrate buffer in a pressure cooker (3 min) was performed. Two washes with H_2_O_2_ (6%) of 10 min each (for CD31, one wash at 3%), followed by a PBS wash with 0.1% Triton was performed to inhibit the endogenous peroxidase. The immunostaining was performed with the Vector Laboratories VECTASTAIN R.T.U. Kit (Newark, CA, USA), using the kit’s normal horse serum for blocking. We then incubated the sample overnight at 4 °C with the primary antibody (anti-CD3 (UCHT1 3 mg/mL; Dako, Santa Clara, CA, USA); anti-CD4 (OTI10B5 a 2 μg/mL; Origene, Rockville, MD, USA); anti-S100A9 + calprotectin (MAC387 1 μg/mL; Abcam, Cambridge, UK); polyclonal anti-CD20 (0.17 μg/mL; Invitrogen, Waltham, MA, USA); polyclonal anti-Ad5 (1.25 μg/mL, Abcam); anti-CD31 (JC70A 1:2, Dako)) and PBS + Triton 0.1% + bovine serum albumin 0.2%. After washing (PBS and Triton 0.1%), we incubated the samples with biotinylated anti-rabbit/mouse secondary antibody (VECTOR, R.T.U. VECTASTAIN Kit) for 30 min. We then incubated it for another 30 min with the kit’s ABC reagent and used the Vector Laboratories’ DAB Peroxidase Substrate Kit to detect the staining. After this procedure, we performed counterstaining with hematoxylin (Harris Hematoxylin solution, PanReac AppliChem, Milan, Italy) and dehydration with alcohol (ethanol 50, 70, 96, 100%, and xylol) before mounting the preparations with dibutylphthalate polystyrene xylene (DPX) medium. Immunolabeling was quantified following a qualitative process by an experienced technician. The whole sample was evaluated using a NanoZoomer^®^ slide scanner at the Instituto de Salud Carlos III over a range of sample sizes, from 2.5 × 0.9 to 23 × 12 mm. Four levels were established: negative (0% positive marking), mild positive (1–25%), moderate positive (26–55%), and high positive (>56%).

### 2.6. Virus-Binding Antibodies

We stored the plasma samples at −80 °C until analysis. The antibodies against canine adenovirus (α-CAV2) were determined by an enzyme-labeled dot assay (Canine VacciCheck, Antibody Test Kit; Eurovet Veterinaria, Madrid, Spain), following the manufacturer’s instructions. We digitized the images and quantified the spot densities with ImageJ software [44]. We calculated arbitrary units as follows: (sample spot intensity–sample mean background intensity)–(positive reference spot intensity–positive reference spot mean background intensity).

### 2.7. Statistical Analyses

We graphed and analyzed the data with GraphPad Prism (GraphPad Software), then performed comparisons between the quantitative variables with a Student’s *t*-test for samples with a normal distribution (* *p* < 0.05).

## 3. Results

### 3.1. Canine Patients, Safety, and Quality of Life

The study included eight oncologic canine patients diagnosed with adenocarcinomas (n = 2; PSit01 and PSit06) and carcinomas (n = 6). Various dog breeds were included: schnauzer (n = 1), labrador retriever (n = 1), golden retriever (n = 1), german shepherd (n= 1), cocker spaniel (n = 1) and mixed breed (n = 3); aged between 7 and 13 years (median 11 years), including females (n = 6) and males (n = 2) (Table 1).

Four patients had been previously treated with chemotherapy and were enrolled in the study when chemotherapy failed: PSit01, PSit02, and PSit07 were treated with palladia, and PSit05 was treated with cyclophosphamide. The rest of the patients’ owners refused other therapies (including surgery when possible), and instead were treated with ICOCAV15 as the first line of treatment. None of them returned to previous treatments after being enrolled in the study. Three patients left the study due to the owners’ request.

**Figure 1:**
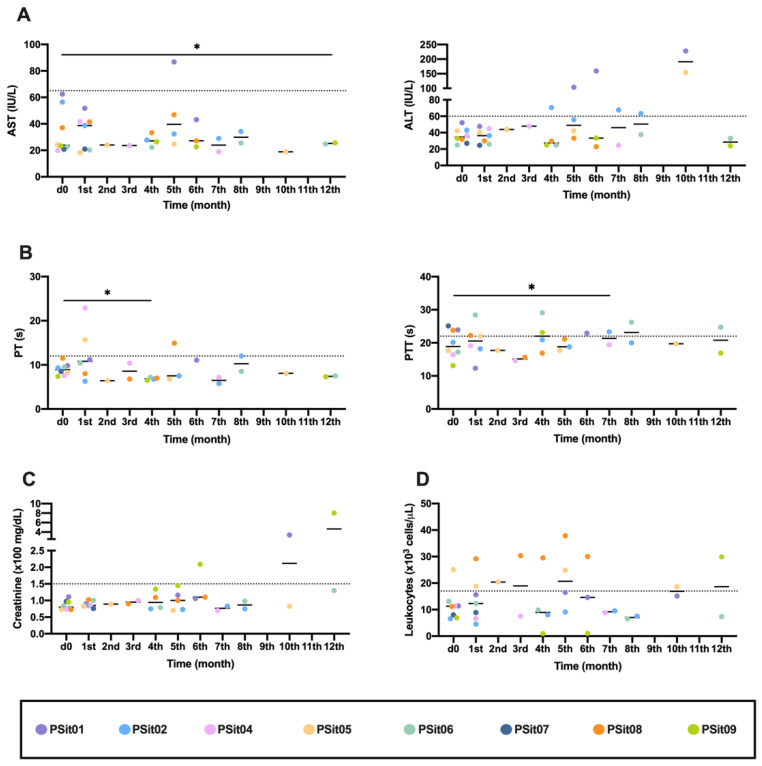
**Hematological and biochemical analysis previously (d0) and during the first year of treatment.** (**A**) Transaminases ALT and AST liver enzymes; (**B**) Clotting times, as PT and PTT measured in seconds (s); (**C**) Creatinine, renal parameters; (**D**) Peripheral blood leukocytes. Individual values of each patient (colored dots) and mean (-) are shown. ALT: alanine transaminase; AST: aspartate transaminase; PT: prothrombin time; PTT: partial thromboplastin time. Dashed line: limit of physiological range. The comparison group for statistical test was d0. * *p* < 0.05.

No generalized change nor changes with clinical relevance were observed in any of the analyzed parameters in the peripheral blood; however, there were particular changes in a few dogs. The canine patients PSit01 (apocrine gland anal sac adenocarcinoma (AGASACA)) and PSit04 (non-tonsillar oral squamous cell carcinoma) had increased hepatic transaminases (Figure 1A). The clotting time was analyzed with blood samples obtained throughout the clinical study, and no clinically relevant alterations were detected (Figure 1B). In the patients PSit01 and PSit09 (breast squamous cell carcinoma (SCC) metastases on the rib wall), we detected an increase in creatinine at months 10 and 12, respectively (Figure 1C). Leukocytes increased after treatment in patient PSit08 (pulmonary adenocarcinoma), although high values were also demonstrated before treatment (Figure 1D).

During follow-up, no abnormalities were detected in the physical exploration by the veterinarian in any of the participants. The questionnaire, performed bimonthly to establish the behavior and quality of life of the patients treated with ICOCAV15, showed that the two dogs diagnosed with pulmonary adenocarcinoma (PSit05 and PSit08) had an increase in the score given by the owners, indicating an improvement in its quality of life. PSit05 was evaluated over 10 months, showing 28, 29, 33, 34, 34 and 26 as average scores, and PSit08 was assessed for 6 months, with average scores of 26, 31, 34 and 33. PSit05, one month after the last score, presented a severe worsening of its quality of life and was euthanized following the owner’s request. However, the virotherapy did not affect the habits of the rest of the patients (six out of eight) (data not shown).

### 3.2. Outcome Assessment of Virotherapy

**Figure 2:**
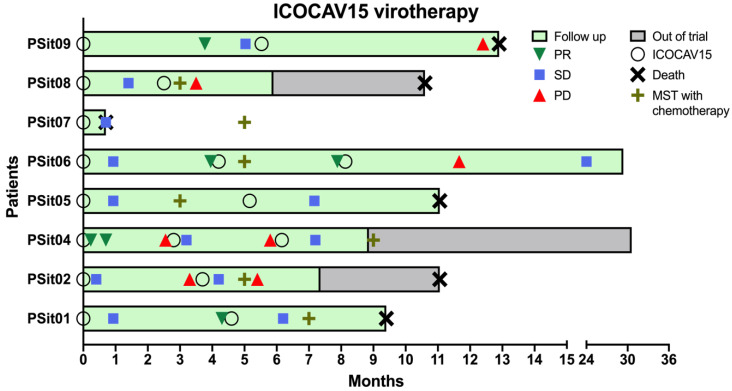
**Median survival time (MST) and outcome assessment of virotherapy.** The time each patient was enrolled in the study (green) or dropped out of the trial (gray), to represent overall survival, is shown. The inoculation of the virus (O) and the outcome assessment following RECIST criteria are represented only if there was a change of category reported. The baseline measurements were taken before each ICOCAV15 dose, and disease evolution was classified as partial response (PR), stable disease (SD), and progressive disease (PD). The patients’ survival time (death; **X**) and the MST with standard of care for each tumor type (**+**) are shown.

After the first inoculation, six dogs presented an SD and two dogs diagnosed with SCC showed PR in the first month and for four months (PSit04 and PSit09, respectively) (Figure 2). It should be mentioned that PSit09 developed a metastasis two months after treatment; nevertheless, the evaluation of the primary lesion showed a good local response from the intratumoral inoculation with ICOCAV15. The patients PSit01 and PSit06 had a PR after 3 months of the initial stabilization, and were both diagnosed with adenocarcinoma. After the second inoculation of ICOCAV15, PSit06 continued to respond to therapy with a PR for 4 months (using the measure taken before the second dose as baseline). A total of four out of seven dogs had an SD after the second dose, and two of them (PSit02 and PSit04) showed a PD after 2 and 3 months, respectively. Two of the patients had a PD at the next revisit, after one and seven months (PSit08 and PSit09, respectively) from the second dose. Lastly, two out of eight patients (PSit04 and PSit06) received a third dose of ICOCAV15, and in both of them an SD was reported to be maintained to date (more than two years after the first dose) (Figure 2).

The overall survival time of the six out of eight (except PSit07 and PSit09) dogs treated with the intratumoral ICOCAV15 was longer than 9 months after administration of treatment. It was compared with the median survival time (MST) according to the reported data in cited references for each case. An acute response, but not sustained, was observed in the patients with squamous cell carcinoma (PSit02 and PSit04). Both patients with adenocarcinoma (PSit01 and PSit06), one with pulmonary adenocarcinoma (PSit05), and the one with rib wall metastases from breast SCC (PSit09) presented a more sustained response over time (Figure 2). The patient PSit09 was maintained with analgesic treatment (tramadol 2 mg/kg twice a day) to control the pain caused by a new metastasis in the pelvis after the first dose of ICOCAV15. Given that there are no specific data for the metastasis of a tumor not previously treated, the MST could not be estimated for patient PSit09.

**Figure 3:**
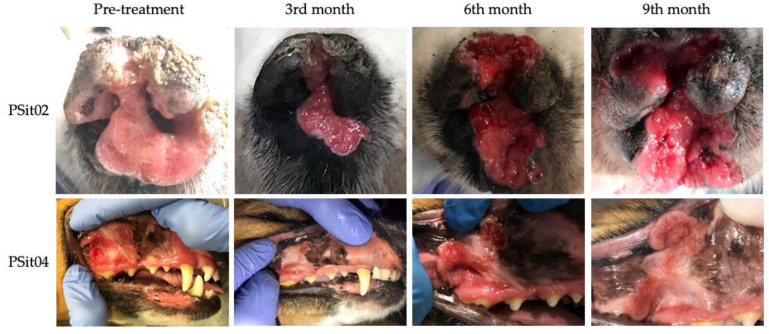
**Images during follow-up from patients diagnosed with squamous cell carcinoma (SCC).** SCC of patient PSit02 in the rostral region of the nasal plane and of patient PSit04 in the buccal region of the right maxillary gingiva. Photographs at medical checkup the day of the pre-treatment (d0) and several months (3rd, 6th, and 9th) after treatment are shown.

**Figure 4:**
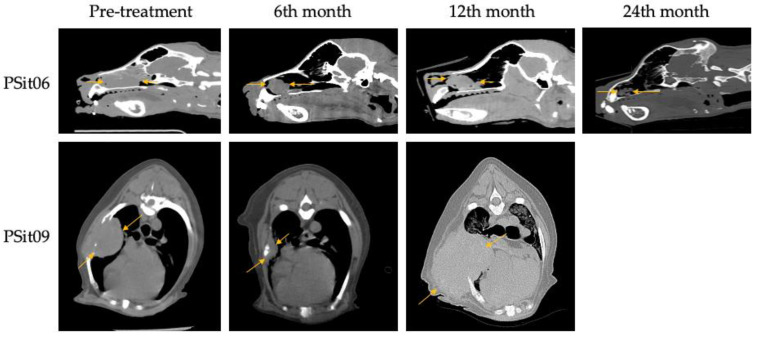
**Computed tomography (CT) images during follow-up.** Nasal adenocarcinoma of patient PSit06 in the nasal cavity and squamous cell carcinoma metastasis in the costal region of patient PSit09 are indicated by arrows. Sagittal slice CT images for patient PSit06 and cross-sectional images for patient PSit09 taken at their largest diameter on the initial day of treatment (d0), at six months (6th), at 12 months (12th), and at 24 months (24th) are shown.

Two patients diagnosed with SCC showed a reduction of 22% (PSit02; baseline sum diameters (BSDs) from 4.5 cm to 3.5 cm) and 61.2% (PSit04; BSD from 6.7 cm to 2.6 cm) of the primary tumor until the third month after treatment (Figure 2 and Figure 3), and patient PSit04 is still alive (2 years and 6 months after the first dose). Then, a progression of the disease was observed and the reinoculation of the virus did not reduce the tumor burden (Figure 2 and Figure 3). Patient PSit06 (nasal adenocarcinoma) had a total reduction of 50.1% (BSD of 14 cm to 6.98 cm) of the primary tumor after the second dose of ICOCAV15. One year after the tumor progressed, but after one additional year without any treatment, the tumor of patient PSit06 was reduced by 50% when compared with its size at the beginning of the therapy (BSD from 14 to 7 cm) and the patient is still alive (2 years and 6 months after the first dose) with an SD (Figure 2 and Figure 4). The patient with a rib wall metastasis from breast SCC (PSit09) achieved a reduction of 48.2% (BSD from 8.1 cm to 4.2 cm) in the sixth month after the first inoculation; a second dose was inoculated once progression started, but a PD was maintained (Figure 4).

### 3.3. Immune Response Evaluation

We have evaluated the immune response from various aspects. The results of flow cytometry tests showed an increase in cytotoxic lymphocytes (CD8+) in the peripheral blood of six out of seven patients after receiving the first dose (the exception was PSit05) (Figure S1).

**Figure 5:**
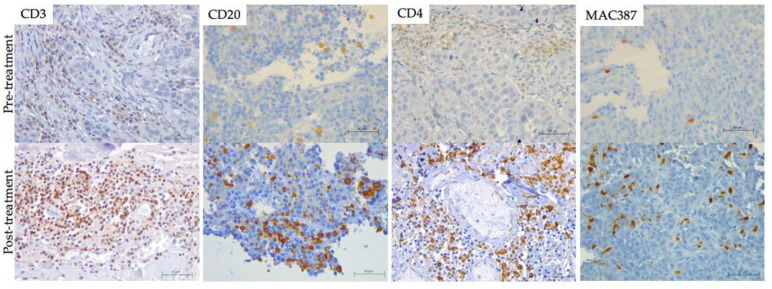
**Tumor Infiltrated immune cells.** (brown) on formalin-fixed paraffin-embedded (FFPE) pre-(**top**) and post-treatment (**bottom**) biopsies including hematoxylin counterstaining (blue). T lymphocytes (CD3) of PSit02 at d0 and d332, T helper lymphocytes (CD4) of PSit06 at d0 and d126, B lymphocytes (CD20) of PSit02 at d0 and d332, and monocytes/macrophages (MAC387) in PSit06 at d0 and d28 are shown. Scale bar: 50 µm.

Regarding cellular infiltration in tissue samples, in patients PSit02 (Figure 5), PSit04 and PSit06 (Figure 5), we observed that the infiltration of T and B lymphocytes (CD3+ or CD20+) increased after the intratumoral inoculation of ICOCAV15. A summary of the results for immune cell infiltrates for each patient are described in Table S2. An increase in lymphocytes was noted after the first dose on day 28, and after the second dose at days 140 and 185 for PSit02 and PSit04, respectively (Table S2). PSit07 and PSit08 (at day 21 and day 42, respectively) showed a reduction in T cell and B cell infiltrates after the first dose (Table S2). PSit05 was the only patient that did not present B lymphocytes in the tumor in any of the samples evaluated. After ICOCAV15 inoculation, the infiltrating monocytes/macrophages increased in five out of eight patients. The highest number of infiltrating MAC387+ was detected after the second dose in PSit02 and PSit04, coinciding with the increase in B and T lymphocytes (Table S2). The endothelial marker CD31 showed that five out of eight dogs were negative when enrolled, and most of them (four out of five) presented low levels at some time during follow-up. Two dogs (PSit06 and PSit07) had reduced endothelial cells, reaching undetectable values at some follow-up time points. Neither moderate nor high amounts of this marker were found in any of the samples.

### 3.4. ICOCAV15 and Antiviral Response

**Figure 6:**
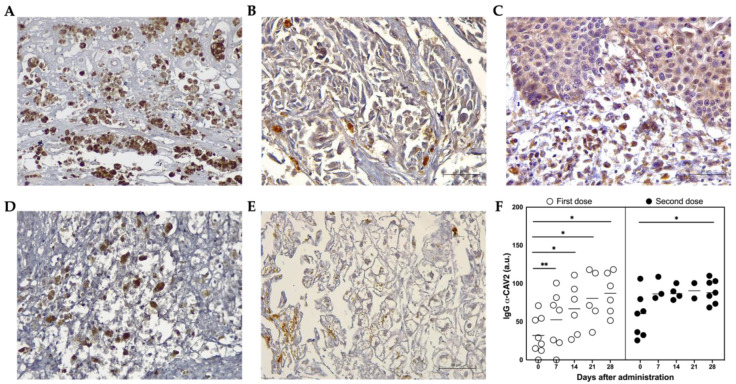
**ICOCAV15 and antiviral response during follow-up.** (**A**–**E**) Representative images of tissues showing adenovirus-positive cells (brown) assessed by immunohistochemistry. Scale bar: 50 µm. (**A**) PSit05 with pulmonary carcinoma 5.8 months after the last OV dose. (**B**) PSit07 with squamous cell carcinoma (SCC) 21 days after the last ICOCAV15 dose. (**C**) Distant metastasis of PSit09 (SCC rib wall) with ICOCAV15 (**D**) Spleen necropsies of PSit05 (pulmonary carcinoma). (**E**) Hepatic tissues of PSit07 (SCC nasal plate). (**F**) IgG α-CAV2 quantified by solid-phase ELISA in peripheral blood is shown. Arbitrary units (a.u.) from each patient (○ and ●) and mean (-) showing anti-adenoviral immunoglobulins present in the serum of canine patients during ICOCAV15 treatment. The comparison group for statistical test was d0. * *p* < 0.05, ** *p* < 0.01.

Adenoviral DNA from ICOCAV15 was detected in tumor biopsies after the first and second inoculation in patient PSit04 by quantitative PCR at days 28 (d28; 828 vp/mg tissue) and 185 (d185; 8731 vp/mg tissue), respectively (Table S2). The presence of ICOCAV15 in treated tumors was confirmed by immunohistochemistry in biopsies (three out of four) during follow-up, and in all evaluated patients at necropsy (five out of five) (Figure 6A,B and Table S2). In some non-treated distant metastases from two dogs (PSit01 in the metastatic liver and PSit09 in the pelvic tumor) ICOCAV15 was also detected (Figure 6C and Table S2). The oncolytic virus was detected in the liver (three out of four) (Figure 6E) and spleen (three out of four) (Figure 6D) of post-mortem patient samples (Table S2). In the month following the first intratumoral inoculation of ICOCAV15, the anti-CAV2 antibodies in peripheral blood were increased in all the patients, despite most of them (seven out of eight) presenting antibodies before treatment. The antibodies increased by 20.7 arbitrary units (a.u.) seven days after the first dose (from 31.9 to 52.6) and increased 25.8 a.u. (from 60.7 to 86.5) after the second dose with the oncolytic virus (Figure 6F).

## 4. Discussion

The use of oncolytic Ads is a promising anti-cancer therapy, showing interesting results in human and veterinary clinical trials. Similar oncolytic Ads loaded into mesenchymal stem cells (Celyvir), used as a new therapeutic approach for canine tumors, have been reported to show a 74% response rate, supporting the hypothesis that CAV could have a beneficial effect on the quality of life of canine oncologic patients [20]. Even though Celyvir shows a good response toward various types of tumors, the direct intratumoral inoculation of the oncolytic Ad ICOCAV15 could improve the oncolytic effect on tumors [20]. It should be noted that, in previous assays, Celyvir had been used as a weekly treatment for several months. In the present study, a maximum of three doses of ICOCAV15 were administered (one dog with one dose, five dogs with two doses, and two dogs with three doses). We believed it would be more effective, given that 90% of the virus had been eliminated intravenously within the first 24 h by elements of the immune system, and even if cell-mediated delivery is a way to avoid such elimination, there is still a risk of it occurring [45,46]. The patients included in the study were only those that had accessible tumors, to avoid administration-associated risks.

The intravenous administration of Celyvir has not demonstrated any negative adverse effects in patients [20]. Intralesional administration ensures the arrival of the virus to the tumor without the animal’s immune system attacking or destroying it before reaching its target. However, a similar study using a modified Ad administered intralesionally reported a case with coagulation deficiencies [38]. To evaluate the safety of ICOCAV15, a test of the dogs’ quality of life, complete examinations, and complete blood tests were performed on our canine patients. In two patients (PSit01 and PSit04), we observed an elevation of the hepatic transaminases after treatment with ICOCAV15, which has not been previously described as a possible adverse effect. PSit04 had an elevated ALT, but the AST enzyme was stable during the entire follow-up. Although ALT is predominant in liver tissue, this is not its only localization; therefore, if there is no increase in both enzymes, ALT should not be associated with liver damage [47]. Patient PSit01 presented a metastatic lesion in the hepatic parenchyma at the time the hepatic transaminases started to elevate. Tumor lesions in the liver have been associated with an elevation of these enzymes, so it is possible that the elevated AST and ALT in PSit01 did not indicate that ICOCAV15 was causing damage to the healthy tissue [47,48]. The elevated creatinine detected in PSit01 and PSit09 was possibly associated with a renal infarction (diagnosed in PSit09) and could have been associated with a paraneoplastic hypercalcemia (PSit01) [7,49]. In addition, considering a previous study from other authors [37], the coagulation parameters were evaluated carefully and frequently, but our dogs did not show any clinically relevant alterations after treatment with ICOCAV15.

The first dose of the oncolytic CAV showed a PR response rate of 25%, and the remaining 75% presented SD. Our results showed that patients with a first response to the treatment maintained a better long-term prognosis for at least 3 months. Furthermore, these patients had a better response rate when they were treated with a second dose of ICOCAV15, even though the progression-free survival (PFS) time was shorter than after the first administration. This differential response to the subsequent doses is well known in immunotherapy, in which tumors can adapt and become resistant to treatment [50]. Combination therapies can be used, and they could possibly improve the PFS in patients with these types of tumors. The MST of the six out of eight dogs treated with the intratumoral virotherapy exceeded the survival time expected for these types of tumors treated with the standard of care protocol (chemotherapy) [2,51,52]. Patients diagnosed with stage III canine AGASACA could be treated with a combination protocol of chemotherapy, but its efficacy has been debated and the MST is approximately 7 months [53]. PSit01 presented an AGASACA with distant metastasis at the time of the first inoculation, and the survival time was 9.5 months, suggesting that adenoviral virotherapy could represent a new protocol with better clinical results and no apparent secondary effects for this type of tumor. Other patients in the study had a highly invasive SCC tumor in the nasal planum. The treatment of choice has been discussed in several articles; given that surgery is aggressive, owners typically reject it, and electrochemotherapy is not considered as the gold standard therapy in dogs. Due to its highly invasive behavior, the MST is approximately 5 months. Our findings suggest that treating these patients with ICOCAV15 improves the survival and the well-being of dogs without the presence of adverse effects [51]. The patients diagnosed with a pulmonary adenocarcinoma, AGASACA, and adenocarcinoma in the nasal planum outlasted the survival time when compared to the MST reported with the standard of care [2]. Given that only four patients in the study met these criteria, more in-depth studies are indicated to confirm this correlation.

We documented a patient (PSit06) that maintained an SD after 29 months from the first treatment. This could have occurred because the viral infection was maintained over time in the tumor, as shown by the detection of OV several months after the last inoculation; furthermore, previous studies have shown that similar OV, when administered intravenously with carrier cells, can be maintained over time in some organs and tissues inside the body [54]. A possible pseudo-progression process could not be ruled out as being responsible for the PD, as has been documented with other immunotherapies [55].

The rising trend of CD8+ lymphocytes in six out of seven dogs 14 days after receiving the first dose was remarkable, given that it was probably due to the release of ICOCAV15 from the tumor into the peripheral blood. This hypothesis appears feasible considering that virus has been detected in other organs and distant metastases that had not been treated. The changes in the other circulating subset frequencies were extremely variable and no conclusions could be drawn.

MAC387 has been identified as a marker for macrophages with an M1-like phenotype, usually associated with a pro-inflammatory and immunosuppressive TME, that favors an antitumoral response [56,57]. Patients with an overall MST higher than that expected with conventional treatment (PSit02 and PSit04) showed an increase in MAC387+ cells in the primary tumor tissue after the administration of the OV, suggesting that ICOCAV15 could have an effect on the TME, further suggesting that the infiltration of these cells could improve survival. In addition, an increase in the infiltration of CD3+ and CD20+ cells has been reported in both patients after the second dose. These data suggest that ICOCAV15 can stimulate the immune environment within the tumor, improving the MST. Even though an increase in the CD3+ and CD20+ immune population within the tumor was reported, a correlation with overall survival time was not found. The infiltration of proinflammatory macrophages and T and B lymphocytes into the tumor after the inoculation of ICOCAV15 should be further investigated as a possible prediction of treatment response in canine patients.

The assays performed in this study demonstrate the presence of ICOCAV15 in distant metastases, which could indeed have an abscopal effect and improve the survival rate for canine patients with unreachable metastasis. In addition, no negative clinical adverse effects, and no negative alterations in the quality of life of patients were reported during the study. It should be noted that PSit09, which developed a distant metastasis with a pelvic tumor after the inoculation of the first dose, presented the virus inside the metastatic tissue after 389 days of treatment; therefore, this finding must be further studied to understand the abscopal effect of ICOCAV15 related to the possible systemic dissemination of the virus. Given that this study was performed on a small number of patients, future studies with a larger population will be necessary to study the effects of treatment with ICOCAV15.

## 5. Conclusions

ICOCAV15 has been shown to be a possible treatment for some carcinomas/adenocarcinomas in veterinary medicine, without any adverse effects, and improving the wellbeing and survival rates in canine oncologic patients. Although the intratumoral inoculation of ICOCAV15 could inhibit growth in distant metastases, this study was performed with a small number of patients; therefore, a more extensive and in-depth study should be performed to confirm these findings.

## Figures and Tables

**Table 1:** Patients’ characteristics.

Patient	Breed	Age (years)	Sex	Diagnosis	ICOCAV15 Doese	Survival Time (days)
PSit01	Miniature Schnauzer	13	Female	Apocrine Gland Anal Sac Adenocarcinoma	2	282
PSit02	Golden Retriever	11	Male	Squamous Cell Carcinoma (nasal plane)	2	332
PSit04	Mixed	7	Female	Squamous Cell Carcinoma (oral)	3	To date (2 years 7months)
PSit05	German Sheperd	10	Female	Pulmonary Carcinoma	2	332
PSit06	Mixed	11	Male	Nasal Adenocarcinoma	3	To date (2 years 7months)
PSit07	Labrador Retriever	13	Female	Squamous Cell Carcinoma (nasal plane)	1	21
PSit08	Mixed	10	Female	Pulmonary Carcinoma	2	319
PSit09	Cocker Spaniel	13	Female	Squamous Cell Carcinoma (rib wall)	2	389

## Data Availability

Not applicable.

## Supplementary Materials

The following supporting information can be downloaded at https://www.mdpi.com/article/10.3390/vetsci9070327/s1; Figure S1: Immune cell phenotype; Table S1: Quality of life questionnaire; Table S2: Tumor infiltrated by immune cells, endothelial cells, and oncolytic virus.
