# Supplementary material for: Safety and Efficacy of an Oncolytic Adenovirus as an Immunotherapy for Canine Cancer Patients
Source: Vet Sci. 2022 Jun 28;9(7):327. doi: 10.3390/vetsci9070327 (PMC9316846; doi:10.3390/vetsci9070327)
Supplement: Supplementary file 1 [file vetsci-09-00327-s001.zip › vetsci-1747817-supplementary.pdf]

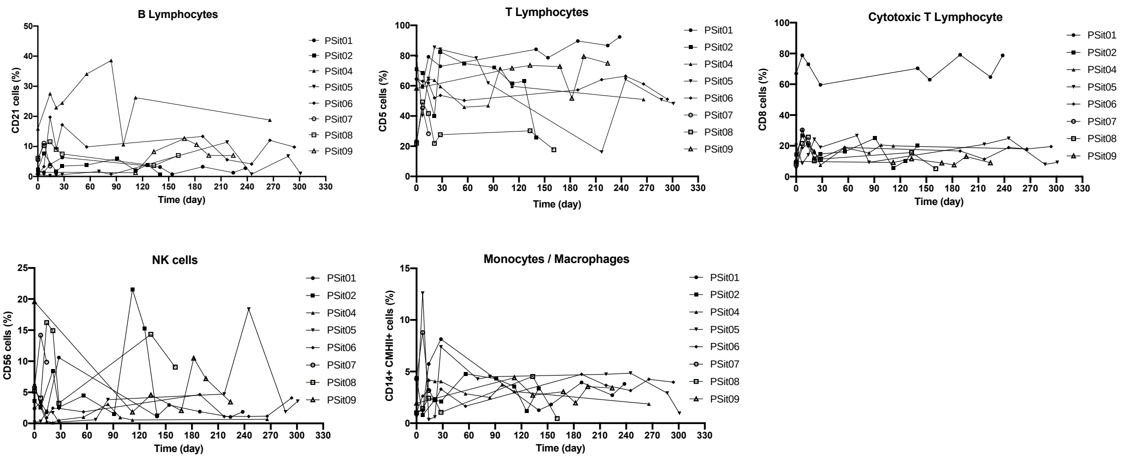

Figure S1. Immune cell phenotype. Immune cell populations in peripheral blood from the day of treatment (d0) and during the first year of follow-up.

Table S1. Quality of life questionnaire. Adapted from [42]

**How much do you think the disease affects your dog´s quality of life?**			
A lot (0)	Quite a lot (1)	A Little (2)	Not at all (3)
**Does he/she still do the activities he/she likes (playing, walking…)?**			
No (0)	Infrequently (1)	Frequently (2)	As usual (3)
**How is your dog´s mood?**			
Altered (0)	Sometimes altered (1)	Slightly changed (2)	Normal (3)
**Does he/she mantain his/her grooming habits?**			
No (0)	Infrequently (1)	Less than before ilness (2)	Yes(3)
**How often do you think your dog experiences pain?**			
All the time(0)	Frequently (1)	Rarely (2)	Never (3)
**Does your pet have appetite?**			
No (0)	Forced or food he/she likes (1)	Less than usual (2)	Normal (3)
**Does your pet get tired easily?**			
Yes (0)	Frequently (1)	Rarely (2)	No (3)
**How would you rate your dog´s rest?**			
Very bad (0)	Bad (1)	Almost normal (2)	Normal (3)
**How often does your dog vomit?**			
Always (0)	Frequently (1)	Rarely (2)	Never (3)
**How would you rate your dog´s intestinal functionality?**			
Very bad (0)	Bad (1)	Almost normal (2)	Normal (3)
**Is your dog able to position himself to urinate and defecate?**			
Never (0)	Rarely (1)	Frequently (2)	Yes (3)
**How much attention does your dog pay to the family?**			
Indifferent (0)	Little attention (1)	Increased attention (2)	No change (3)

Table S2. Tumor infiltrated by immune cells, endothelial cells, and oncolytic virus. ICOCAV15 detected by qPCR (#) or IHC. *ICOCAV15 administered. M: metastatic tissue. N/A: not assessed.

			CD3	CD4	CD20	MAC387	CD31	ICOCAV15
PSit01	Necropsy (d282)	Tumor	Medium	Negative	Low	Low	Negative	Positive
		Lung (M)	N/A	N/A	N/A	N/A	N/A	Negative
		Liver (M)	N/A	N/A	N/A	N/A	N/A	Positive
		Spleen	N/A	N/A	N/A	N/A	N/A	Negative
PSit02	Tumor	d0 *	Medium	Negative	Medium	Medium	Negative	N/A
		d28	High	High	High	Medium	Negative	Positive
		d112 *	Medium	Medium	Medium	Medium	Negative	Negative
		d140	High	Medium	High	High	Low	Negative
		d221	Medium	High	High	High	Low	Positive
	Necropsy (d332)	Tumor	High	Medium	High	High	Low	Positive
		Liver	N/A	N/A	N/A	N/A	N/A	Positive
		Spleen	N/A	N/A	N/A	N/A	N/A	Positive
PSit04	Tumor	d0 *	Low	Low	Medium	Low	Negative	N/A
		d28	High	Medium	High	Medium	Low	Positive #
		d84 *	Medium	Medium	Medium	Medium	Low	Negative
		d185 *	High	Medium	High	High	Low	Positive #
PSit05	Necropsy (d332)	Tumor	Medium	Low	Negative	High	Low	Positive
		Liver	N/A	N/A	N/A	N/A	N/A	Negative
		Spleen	N/A	N/A	N/A	N/A	N/A	Positive
PSit06	Tumor	d0 *	High	Low	Medium	Low	Negative	N/A
		d28	High	Low	Medium	Medium	Low	Positive
		d126 *	High	Low	High	Negative	Negative	Positive
		d244 *	Low	Medium	Negative	Low	Negative	Positive
		d350	High	Medium	Medium	Low	Low	Positive
PSit07	Tumor	d0 *	High	Medium	Medium	Low	Low	N/A
	Necropsy (d21)	Tumor	Medium	Medium	Negative	Medium	Negative	Positive
		Liver	N/A	N/A	N/A	N/A	N/A	Positive
		Spleen	N/A	N/A	N/A	N/A	N/A	Positive
PSit08	Tumor	d0 *	High	Low	Medium	Low	Negative	N/A
		d42	Low	Low	Negative	Low	Negative	Negative
PSit09	Tumor	d0 *	High	High	Low	Low	Negative	N/A
	Necropsy (389)	Pelvic tumor (M)	Medium	Medium	Low	Medium	Low	Positive
		Ribwall tumor	Low	Medium	Low	Medium	Negative	Positive
